# Effect of myo-inositol supplementation in mixed ovarian response IVF cohort: a systematic review and meta-analysis

**DOI:** 10.3389/fendo.2025.1520362

**Published:** 2025-03-21

**Authors:** Jianeng Zhang, Huanhuan Zhang, Wenjing Zhou, Meiyan Jiang, Xianhua Lin

**Affiliations:** Reproductive Center, Hangzhou Women’s Hospital, Hangzhou, China

**Keywords:** myo-inositol, *in vitro* fertilization embryo transfer, polycystic ovary syndrome, poor ovary responder, outcomes

## Abstract

**Objective:**

There has been substantial research conducted recently on the effect of myo-inositol (MI) on human reproduction. However, it still remains ambiguous about the therapeutic efficacy of MI in infertile women undergoing *in vitro* fertilization embryo transfer (IVF-ET). This systematic review and meta-analysis was carried out to investigate the efficacy of MI on IVF outcomes.

**Methods:**

Literatures were searched in the PubMed, Web of Science, Cochrane Library, ScienceDirect and Wanfang databases. The methodological quality was assessed using the Cochrane Risk of Bias tool. Data were pooled using a random- or fixed-effects model according to study heterogeneity. The results are expressed as odds ratio (OR) or mean difference (MD) with 95% confidence intervals (CIs). Heterogeneity was measured by the I^2^ statistic. The protocol was prospectively registered with PROSPERO (CRD42024582149).

**Results:**

Eleven eligible studies with 981 participants reported the IVF outcomes of the MI group versus the control group. The synthesis results showed that the metaphase II (MII) oocyte rate was higher in the MI group than in the control group (OR 1.55, 95% CI 1.04-2.31, *P*=0.03). For polycystic ovary syndrome (PCOS) women, as well as non-obese PCOS women, a statistically significant improvement in MII oocyte rate were assumed after taking MI (OR 1.97, 95% CI 1.20-3.25, *P*<0.01; OR 1.92, 95% CI 1.09-3.37, *P*=0.02) while there is no statistically significant advancement showed in the poor ovary responder (POR) women(OR 0.97, 95% CI 0.35-2.68, *P*=0.95). The fertilization rate was higher in the MI group than in the control group (OR 1.62, 95% CI 1.21-2.16, *P*<0.01), for PCOS, non-obese PCOS and POR women (OR 1.59, 95% CI 1.16-2.18, *P*<0.01; OR 1.87, 95% CI 1.52-2.31, *P*<0.01; OR 2.42, 95% CI 1.48-3.95, *P*<0.01).

**Conclusions:**

Our results suggest that MI supplementation improves the MII oocyte rate and the fertilization rate. More high-grade evidence from prospective randomized studies is warranted.

**Systematic review registration:**

https://www.crd.york.ac.uk/PROSPERO/, identifier CRD42024582149.

## Introduction

IVF-ET is supposed to help infertile couples to achieve pregnancy (1). With the breakthrough of novel technologies in ovulation induction protocols and laboratory, the successful rate in IVF has progressively risen (2). However, there is still a considerable number of infertile couples who cannot conceive through IVF. Improving the quantity and quality of embryos is a crucial factor contributing to the success of IVF-assisted reproduction (3–5). Great efforts have been made to discover adjuvant therapy or supplementation attempting to get ideal IVF outcomes (6, 7).

As a supplementation in IVF, inositol has attracted increasing attention. It is a compound that occurs naturally in many foods and is an essential component of the vitamin B group (8). Studies have shown a correlation between the level of inositol in follicular fluid and the quality of oocytes (9). It was also confirmed that women who achieved pregnancy through *in vitro* fertilization and embryo transfer had higher levels of inositol in their follicular fluid than non-pregnant women (10). MI, one of the nine different forms of inositol, can be converted into inositolphosphoglycan within the human body, which acts as a secondary messenger involved in insulin signal transduction, primarily regulating the activation of glucose transporters and glucose utilization (11, 12). Therefore, its function as an insulin sensitizer is used to treat PCOS of which insulin resistance is one of the typical symptoms (13–16). Although researches conducted to investigate the effectiveness of inositol on PCOS women attending intracytoplasmic sperm injection (ICSI) programs indicated no significant improvement on the quality of oocyte or embryo or pregnancy rates, the combination use of d-chiro-inositol (DCI), another form of inositol, left the conclusion undetermined, because DCI was reported to act differently with MI may even have a negative effect on oocyte quality (17, 18). Moreover, MI is also utilized in non-PCOS women in IVF, including normal responders and poor responders (19, 20), since it plays a crucial role in cell growth and follicle-stimulating hormone (FSH)signal transduction (21, 22), which is associated with oocyte maturation and embryo development.

At present, the data regarding the therapeutic efficacy of MI in infertile women undergoing IVF is still rather unclear. Thus, this systematic review and meta-analysi**s** aims to systematically review and summarize the evidence examining the impact of MI on IVF outcomes to further optimize clinical treatment strategy.

## Materials and methods

### Search strategy and study selection

The growing number of observations have focused on the effect of MI in assisted reproductive technologies since the concentration of MI in the follicular fluid was found directly correlates with the quality of oocytes and embryos in 2002 (9). In this systematic review, we searched for studies published in the last two decades until July 2024 in the following databases: PubMed, Cochrane Library, Web of Science, Science Direct, and WANFANG Database. A combination of MeSH terms and free words were used. The main search terms were ‘myoinositol’ or ‘inositol’ and ‘IVF’ or ‘*in vitro* fertilization’ or ‘*ICSI*’. The language was restricted to English and Chinese in the searches. A hand-search of reference lists of the included studies or relevant recent reviews was conducted to identify potential data resources.

**Figure 1:**
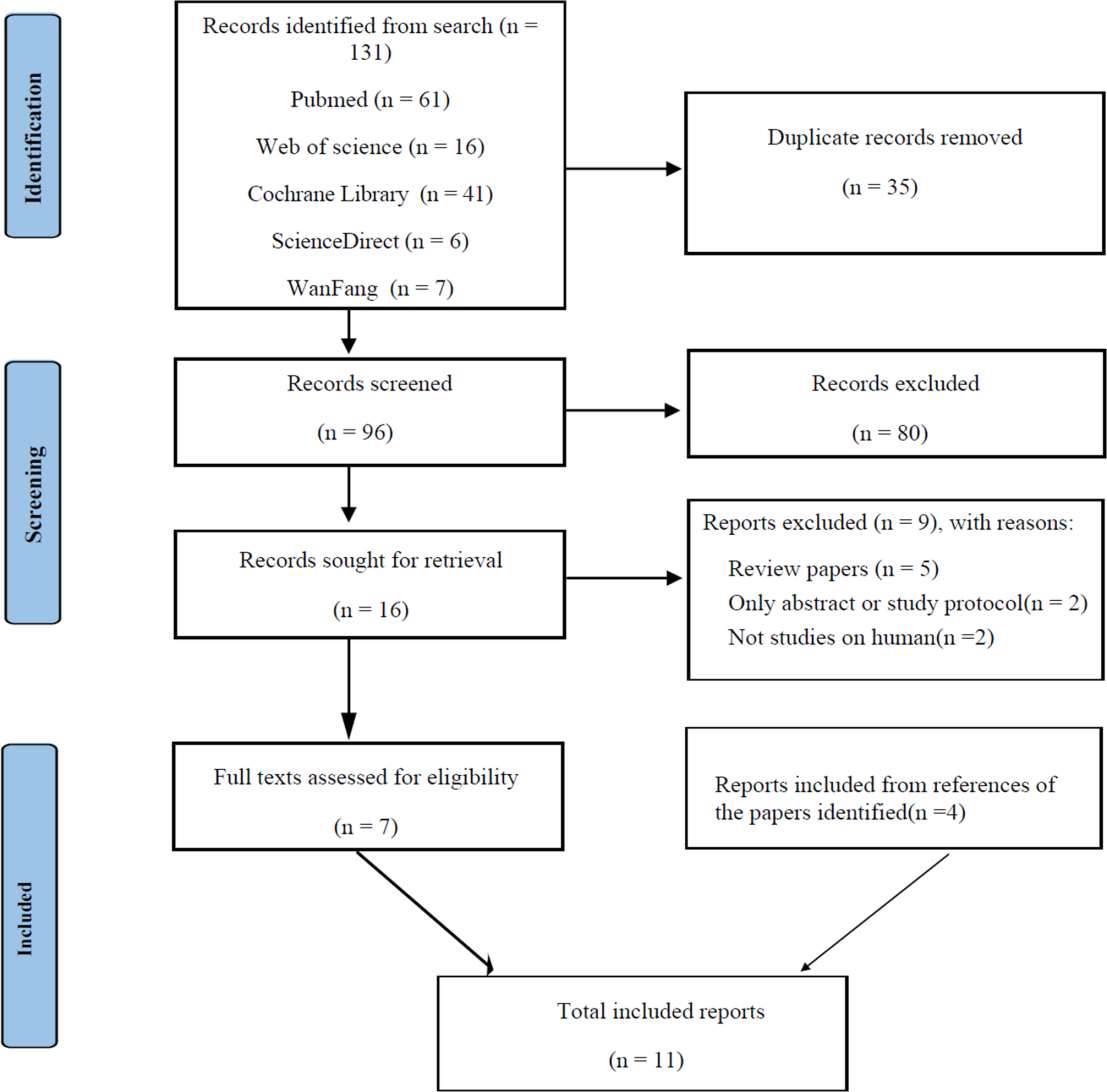
PRISMA diagram for literature search process and total number of studies screened and included at each stage.

Titles and abstracts were screened independently in duplicate by 2 reviewers, and disagreements were resolved by discussion, with a third reviewer to adjudicate if needed. The identified studies were reviewed to be included or excluded by 1 reviewer and verified by the second reviewer. The study selection process for the systematic review is shown in **Figure 1**.

### Inclusion criteria

We included randomized controlled trials and controlled observational studies that compared IVF outcomes between MI adjuvant therapy and usual care or placebo. The inclusion criteria were as follows: (1) female participants diagnosed with infertility, undergoing IVF or ICSI. (2) with MI adjuvant therapy being the main difference between the intervention group and the control group.

### Exclusion criteria

In this meta-analysis, we excluded studies published only as abstracts or repeated publications, as well as studies with co-interventions used, except folic acid, as the effect of folic acid (FA) on outcomes was thought to be minimal.

### Study appraisal and data extraction

The methodological quality of all the selected studies was assessed by two reviewers independently. For randomized studies, quality assessment was performed by following Cochrane Handbook for Systematic Reviews of Interventions, information on the randomization method, allocation concealment, blinding, intention-to-treat analysis and follow-up rate was assessed (**Supplementary Table S1**). The Newcastle-Ottawa Scale (NOS) was used to evaluate the methodological quality of the three non-randomized studies (**Supplementary Table S2**). Any disagreements were resolved by discussion.

For each study, data obtained from the manuscript included the first author, year of publication, country of origin, study design, participant characteristics and intervention protocol, etc. All the data were extracted by one researcher and verified by a second researcher.

### Statistical analysis

This meta-analysis was performed using Review Manager 5.4.1 (23). Heterogeneity was assessed using the chi-squared and I^2^ tests, with significant heterogeneity defined as P < 0.05 or I^2^ > 40%. Random effects models were adopted when P< 0.05 or I^2^>40%; otherwise, fixed-effects models were used. For continuous data, MD with 95% CI was used to express the effect estimate, while pooled OR with 95% CI was used for categorical data. Subgroup analysis was conducted according to the work-up of the participants (PCOS/POR) if there was a difference among the population for one individual indicator between studies. In addition, a sensitivity analysis was conducted to examine the heterogeneity and differences in outcomes as well as publication bias using funnel plots to assess the bias.

## Results

### Characteristics of included studies

A total of 131 articles were identified by the literature search. First, 35 duplicate articles were removed. Initial screening of the titles and abstracts excluded 80 unsuitable articles, and 7 articles remained after reviewing the full text of the identified articles. In addition, 4 articles were retrieved from the references of the identified articles.

Finally, eleven studies were included in this review and meta-analysis. Among them, seven studies reported the IVF outcomes of the MI group versus the control group in PCOS participants, three studies reported the outcomes in POR participants and the remaining reported the results in non-PCOS participants. The pooled sample size was 981 (478 in the observation group and 503 in control group). The study participants were mainly from Europe, the Middle East, and Eastern Asia. The ages of the participants, as well as the study interventions, are presented in **Table 1**.

**Table 1:** Characteristics of the 11 studies included in the meta-analysis.

Author	Year	Country	Study design	Population	Sample size (n)		Age(mean ± SD)		Protocols	
					Observation	Control	Observation	Control	Observation	Control
Sene	2019	Iran	RCT	women with PCOS, undergoing IVF,BMI<28	25	25	31.3 ± 4.1	29.8± 4.5	4 g MI combined with 400 mg FA,1 month before starting the antagonist cycle until the day of ovum pick up	400 mg FA
Lesoine	2016	Germany	RCT	women with PCOS, aged<40 years, undergoing IVF	14	15	_	_	MI 4 g/day and FA 400 µg/day, 2 months prior to IVF cycle	placebo
Nazari	2019	Iran	RCT	poor responders, undergoing ICSI	56	56	38.0 ± 4.6	37.7 ± 4.4	MI 4 g and FA 400 mg daily from 1 month before starting the ICSI cycle continuing until the ovulation triggering day	FA 400 mg daily
Caprio	2015	Italy	controlled observational trial	poor-responders, undergoing ICSI	35	30	33.2 ± 2.8	33.9 ± 3.1	4 g of MI+ 400 μg of FA daily, previous 3 months before the enrollment day	400μg of FA
Mohammadi	2021	Iran	RCT	poor-responders, undergoing ICSI	30	30	35.0 ± 6.9	36.7 ± 5.6	4 g MI + 400 μg FA for 12 weeks before the enrollment day	FA 400 μg
Lisi	2012	Italy	prospective, randomized, open-label, pilot study	non-PCOS women, undergoing IVF	50	50	34.4 ± 3.4	33.3 ± 2.8	4g MI daily + 400 μg of FA in the 3 months before and through treatment	400 μg of FA
Papaleo	2009	Italy	RCT	women with PCOS, undergoing ICSI, BMI<28	30	30	36.2 ± 2.4	35.4 ± 2.5	MI combined with FA 2 g twice a day, starting on the day of GnRH administration	FA alone
Ciotta	2011	Italy	RCT	Women with PCOS, aged <40 years, undergoing IVF	17	17	_	_	2 g of MI + 200 mg of FA, twice a day, continuously for 3 months.	200 mg of FA
Kitaya	2019	Japan	prospective controlled observational study	women with PCOS undergoing the first ICSI cycle, BMI<28	25	25	32.1 ± 3.7	31.6 ± 4.1	4 g/day MI + 400 μg/day FA supplementation, initiated on day 3 of the cycle	400 μg/day FA
Tabatabaie	2022	Iran	RCT	PCOS, candidates for IVF cycles, BMI<28	30	30	29.6± 3.5	31.2 ± 3.1	a daily dose of 4 g MI combined with 400 mg of FA, from 1 month prior to IVF cycle until the day of ovum pick up	daily dose of 400 mg of FA
Pacchiarotti	2015	Italy	RCT	PCOS, undergoing IVF, BMI<28	166	195	31.5 ± 2.8	32.0 ± 3.6	MI 4g and FA 400mg	FA 400mg

PCOS, polycystic ovary syndrome; ICSI, intracytoplasmic sperm injection; IVF, *in vitro* fertilization; MI, myo-inositol; RCT, randomized controlled trial; FA, folic acid; BMI, body mass index.

### Quantitative data synthesis

The statistical results of the comparison between the MI group and the control group are presented in **Table 2**, and the forest plots are shown in **Supplementary Figures S1**-**S5**.

**Table 2:** Summary of results of meta-analysis of comparison between obsevrvational and control group.

Outcome indicator	Studies	Samples	Heterogeneity	Effect model	MD/OR (95%CI)	*P* value
NO. oocytes retrieved	10	952	I^2^ = 61% P=0.006	Random	0.22[-0.43, 0.88]	0.50
PCOS	6	615	I2 = 53% P=0.06	Random	0.84 [-0.54, 2.22]	0.23
Non-obese PCOS	5	581	I2 = 0% P=0.64	Fixed	0.27 [-0.14, 0.69]	0.20
POR	3	237	I2 = 26% P=0.26	Random	0.29 [-0.29, 0.88]	0.33
MII oocyte rate	10	6528	I^2^ = 91% P<0.01	Random	1.55 [1.04, 2.31]	0.03*
PCOS	6	5200	I^2^ = 94% P<0.01	Random	1.97 [1.20, 3.25]	<0.01*
Non-obese PCOS	5	4747	I^2^ = 95% P<0.01	Random	1.92 [1.09, 3.37]	0.02*
POR	3	653	I2 = 76% P=0.02	Random	0.97 [0.35, 2.68]	0.95
Fertilization rate	9	4151	I^2^ = 75% P<0.01	Random	1.62 [1.21, 2.16]	<0.01*
PCOS	5	2998	I^2^ = 74% P<0.01	Random	1.59 [1.16, 2.18]	<0.01*
Non-obese PCOS	4	1884	I^2^ = 35% P=0.22	Fixed	1.87 [1.52, 2.31]	<0.01*
POR	3	543	I2 = 21% P=0.28	Random	2.42 [1.48, 3.95]	<0.01*
High quality embryo rate	3	715	I^2^ = 81% P<0.01	Random	1.54 [0.48, 4.93]	0.46
Clinical pregnancy rate	4	334	I^2^ = 0% P=0.76	Fixed	1.53 [0.93, 2.53]	0.09

MD, mean difference; OR, odds ratio; CI, confidence interval; *P<0.05.

### Oocytes retrieved

Ten studies (19, 24–32), including reported the Number (No.) Sof oocytes retrieved. Six studies (27–32) involved the PCOS group, three studies (24–26) involved the POR group and the remaining one (19) involved the non-PCOS participants. Pooling of the results from the ten studies did not show a statistically significant difference in No. Oocytes retrieved between the MI group and the control group (MD 0.22, 95% CI -0.43-0.88, *P* = 0.5; **Table 2**). Meanwhile, there was no statistically significant improvement in No. oocytes retrieved in the MI group compared with the control group for PCOS participants (MD 0.29, 95% CI -0.29-0.88, *P*=0.33; **Table 2**). In addition, 5 studies (27–30, 32) on PCOS reported women enrolled were not obese, the meta-analysis still showed no significant difference in No. oocytes retrieved (MD 0.27, 95% CI -0.14-0.69, *P* = 0.2; **Table 2**). The data for POR participants presented no significant improvement, either (MD 0.84, 95% CI -0.54-2.22, *P*=0.23; **Table 2**).

### MII oocyte rate

Ten studies (19, 24–32) reporting MII oocyte rate showed a statistical significance in the advancement in the MI group (OR 1.55, 95% CI 1.04-2.31, *P*=0.03; **Table 2**). Six studies (27–32) involving the PCOS group assumed a statistically significant improvement in MII oocyte rate after taking MI (OR 1.97, 95% CI 1.20-3.25, *P*<0.01; **Table 2**), and the significant improvement also showed in the non-obese women (27–30, 32)in this subgroup analysis (OR 1.92, 95% CI 1.09-3.39, *P*=0.02; **Table 2**). However, there is no statistically significant advancement showed in the POR subgroup (OR 0.97, 95% CI 0.35-2.68, *P*=0.95; **Table 2**) (25–27).

The heterogeneity test for the data synthesis showed that the χ^2^ value was 1.51, with df =1 and *P*=0.22, while I^2^ was 33.9%, suggesting no statistical heterogeneity among the included studies between the subgroups.

### Fertilization rate

Nine studies (19, 24–28, 30, 32, 33) reported fertilization rate. The test for overall effect showed a statistically significant improvement in the fertilization rate in the MI group compared with the control group (OR 1.62, 95% CI 1.21-2.16, *P*<0.01; **Table 2**). The five studies, involving the PCOS subgroup (27, 28, 30, 32, 33), also showed a statistically significant improvement in the fertilization rate after taking MI (OR 1.59, 95% CI 1.16-2.18, *P*<0.01; **Table 2**). The significant improvement was also observed in the non-obese PCOS women (OR 1.84, 95% CI 1.41-2.40, *P*<0.01; **Table 2**). Likewise, the three studies involving the POR subgroup (24–26) showed a statistically significant difference in the fertilization rate between the MI group and the control group (OR 2.42, 95% CI 1.48-3.95, *P*<0.01; **Table 2**).

The heterogeneity test for the data synthesis showed that the χ^2^ value was 2.0, with df=1 and *P*=0.16, while I^2^ was 49.9%, suggesting there is statistical heterogeneity among the included studies between the subgroups.

### Cleavage rate

Two studies (19, 30) reported cleavage rate. Lizi F, et al. reported there was 171 out of 202 fertilized oocytes grow to cleavage, and Papaleo E, et al. reported 149/169 after using MI.

### High-quality embryo rate

Three studies (19, 30, 31) comparing the high-quality embryo rate between the MI group and the control group were included in the data synthesis. The results showed no significant difference between the two groups under a random-effects model (OR 1.54, 95% CI 0.48-4.93, *P*=0.46; **Table 2**).

### Blastocyst rate

Only one study (27) reported on blastocyst rate, which indicated that there were 89 blastocysts cultivated out of 206 cleavage embryos.

### Implantation rate

There were three studies (19, 25, 26) reporting on implantation rate, but only Lisi et al. (19) supplied the raw data, 21 out of 112 (18.7%) embryos implanted in the MI supplement group. In the other two studies (25, 26), the implantation rate was 7.94% and 10.8%.

### Clinical pregnancy rate

Four studies (19, 24, 29, 30) reported the clinical pregnancy rate. The test for overall effect showed no statistically significant improvement in the clinical pregnancy rate after taking MI (OR1.53, 95% CI 0.93-2.53, *P*=0.09; **Table 2**). Furtherly, Pacchiarotti et al. reported 63.3% clinical pregnancy rate, and Papaleo et al. reported 8 out of 30 got pregnancy after using MI when PCOS women undergoing IVF procedure.

### Publication bias

**Figure 2:**
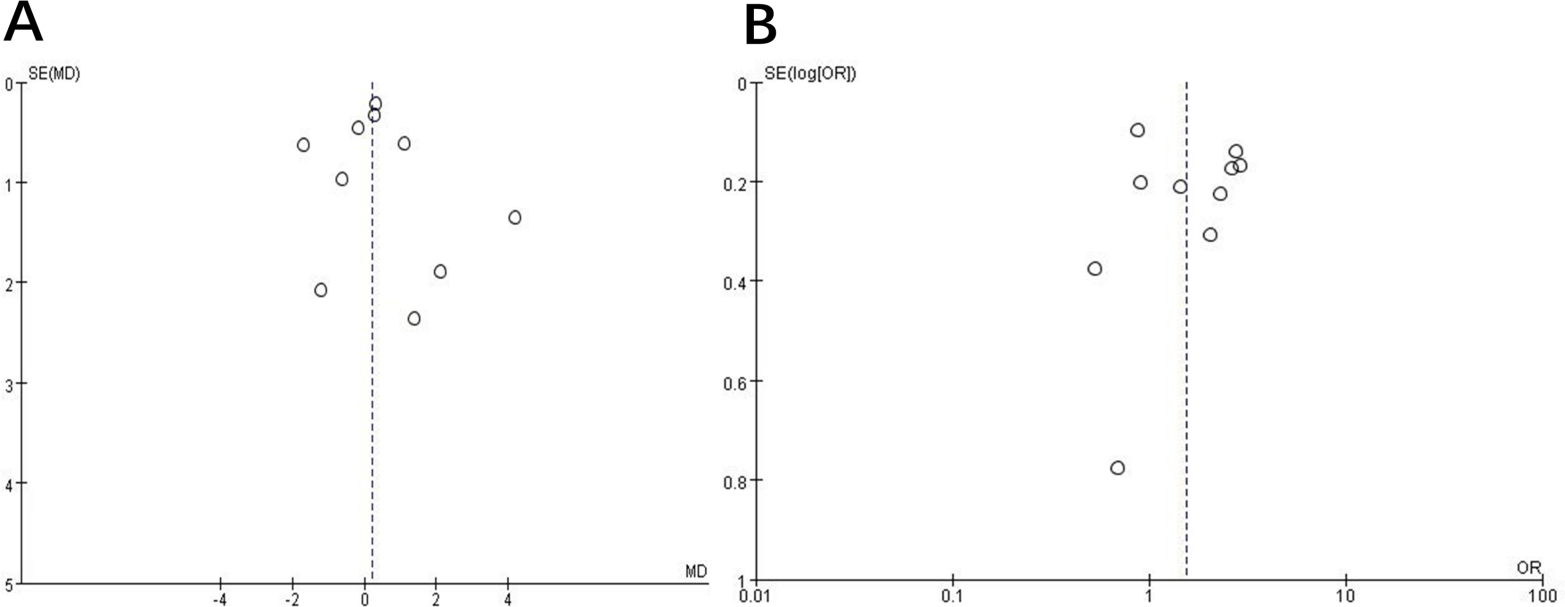
Funnel plot to assess publication and related biases in the systematic review. **(A)** continuous data; **(B)** categorical data.

Publication bias was assessed using funnel plots. The analysis results for publication and related bias did not suggest evidence of bias (**Figure 2**).

### Sensitivity analysis

Generally, if the I^2^ test results exceed 40%, the heterogeneity is considered high. Consequently, a random-effects model is employed for analysis when I^2^ surpasses 40%; otherwise, a fixed-effects model is utilized. Sensitivity analysis was conducted by sequentially excluding individual studies. Statistically similar results were obtained for each indication except after excluding study of Sene or Tabatabaie while evaluating the sensitivity for the MII oocyte rate in non-obese PCOS women. Overall, the result showed statistical significance (OR 1.92, 95% CI 1.09-3.39, *P*=0.02), but it showed no significant improvement (OR 1.78, 95% CI 0.91, 3.47, *P*=0.09; OR 1.74, 95% CI, *P*=0.10) after deleting these two studies separately despite I^2^ sustained at 95%, *P*<0.05. This was likely due to the range of sample sizes. Therefore, data from this systematic analysis should be interpreted with caution until further high-grade evidence emerges.

## Discussion

Increasing evidence has emerged to support the use of inositol in human reproduction, particularly for women with PCOS, but its effectiveness on IVF outcomes remains uncertain. This meta-analysis found that pre-treatment with MI may improve the MII oocyte rate in women undergoing IVF. This improvement remained in the PCOS subgroup as well as the non-obese PCOS women, but not in the POR subgroup. In addition, the positive impact on fertilization rate was consistent among the PCOS, the non-obese PCOS and POR subgroups, although the cleavage rate, high-quality embryo rate, or clinical pregnancy did not improve significantly (34). The results of sensitivity analysis by excluding studies with the most extreme dose variation (MI 2g daily) indicated that the variable dose of MI did not unduly influence the results. The effect of myoinositol supplementation on IVF outcomes in PCOS women with insulin resistance was a predetermined outcome. However, it failed to be analyzed since the studies on PCOS either excluded patients with diabetes or hyperinsulinemia, or did not provide data on insulin resistance.

Studies have indicated a significant positive correlation between MI concentration in follicles and various reproductive factors such as estradiol level in follicular fluid, cleavage rate of fertilized oocytes, embryo stage (± 4 cells), and embryo quality (grade) (9). A higher concentration of inositol in human follicular cells serves as a biological indicator for the improvement of oocyte quality (9, 35). Inositol can exist within cells in a free form or as a binding component of phospholipids or inositol phosphate derivatives (such as inositol triphosphate, etc.) in the plasma membrane, where they are crucial for cell growth, insulin, and FSH activity (36). Inositol serves as a precursor of inositol phosphate, which is a key component of the phosphatidylinositol signal transduction system. This signal transduction system involves the hydrolysis of phosphatidyl-inositol bisphosphate-dependent receptors to generate two important second messengers: trisphosphate (InsP3) and diacylglycerol (DAG) (37). InsP3 diffuses into the cytoplasm and binds to inositol InsP3 receptors on the surface of the endoplasmic reticulum, leading to the release of intracellular calcium oscillations. DAG activates protein kinase C, which modulates various cellular processes such as gamete formation, fertilization, cell proliferation, and development by phosphorylating proteins in diverse cell types (38, 39). Inositol performs various functions at the ovarian level, particularly the role of InsP3 in regulating intracellular calcium concentrations in response to the effects of the luteinizing hormone and FSH (40, 41). The function of this molecule seems to be crucial in the maturation of oocytes (42).

As in our research, MI was reported to contribute to the quality and maturity of oocytes, the cleavage rate, blastocyst expansion, and embryo quality (43). Nevertheless, it has been also demonstrated that in women with PCOS, MI was insufficient to improve the MII, embryo quality or pregnancy rate in women with PCOS (17). In this study, any dose and duration of inositol pretreatment, either MI or DCI, were included. Currently, the data on the effects of DCI on the ovary is inconsistent. The usual ratio of MI/DCI in the follicular fluid is 100:1 but is altered to 0.2:1 in PCOS patients (16). A decrease in MI concentration may lead to excessive DCI due to epimerase overactivation, resulting in depleted MI levels and subsequent decline in embryo quality (11). The dosage, duration of supplementation, and the ratio of two types of inositol are likely influencing factors on its effects which warranted further evaluation by large-sample randomized controlled trial (RCT) studies. Meanwhile, there was evidence showing that increasing the dose of DCI gradually deteriorated oocyte quality and the total amount of r-FSH (18). Therefore, this meta-analysis excluded studies with interventions of DCI supplementation or combination with metformin to ensure the accuracy of the data. Only studies with MI supplementation were included.

MI may enhance oocyte responsiveness to intracellular calcium oscillations during the early stages of fertilization, thereby potentially improving fertilization rate and embryo quality (25). It plays a role in the release of cortical granules, inhibition of polyspermy, meiosis, and subsequent activation of the cell cycle (19). In a mouse model, elevated levels of MI lead to an increase in intracellular calcium oscillations and the end of meiosis (44). The exposure of fully grown mouse germinal vesicle oocytes to MI during *in-vitro* maturation can enhance meiotic maturation, and the subsequent developmental potential of these oocytes following fertilization. Colazingari G et al. found that MI supplemented embryos displayed a faster cleavage rate and by the end of preimplantation development, the majority of MI supplemented blastocysts was expanded and formed by a higher number of blastomeres (45). Mohammadi F et al. showed that MI could decrease Intracellular reactive oxygen species and increase glutathione and mitochondrial membrane potential levels and consequently prevent oocyte quality reduction and improve fertilization potential in mouse (46). In humans, it has been demonstrated that the incidence of gestational diabetes mellitus was significantly reduced in women supplemented with MI. Moreover the incidence of fetal macrosomia (birth weight > 4000 g), gestational hypertension, preterm delivery, caesarean section, shoulder dystocia, neonatal hypoglycemia, and neonatal transfer to an intensive care unit) did not reveal appreciable differences between the MI and the placebo‐exposed groups (47).

In the present study, the MII oocyte rate did not increase in women with POR, which may be attributed to the multifactorial nature of oocyte quality, encompassing nuclear and mitochondrial genomes, as well as the ovarian and follicle microenvironments influencing cytoplasmic maturation, with inositol being just one contributing factor (48).

Achieving clinical pregnancy not only relies on high-quality embryos but also requires an endometrium with good receptivity, normal immune and coagulation functions as well as consideration for the psychological state of the infertile women. Improving clinical pregnancy rates demands multiple intervention measures rather than relying solely on one method since any abnormality at any stage may result in failed embryo implantation.

Overall, data from this systematic review should be interpreted with caution because of the limitations. Firstly, the robustness of the results depends largely on the quality of the primary studies included in this review. Inclusion of both RCTs and observational studies might introduce methodological heterogeneity in some instances. Meanwhile, adverse events were not reported by the majority of studies. Secondly, substantial disparities in patient selection and the variability in sample sizes decreased the certainty of the evidence overall as women with PCOS and POR accounted for a large proportion of participants in this meta-analysis. Significant statistical heterogeneity (I² >50%) in outcomes, might invalidate fixed-effect models, necessitating cautious interpretation of random-effects results. Consequently, the data derived from this systematic analysis should be interpreted with caution until further high-quality evidence becomes available. Future studies should standardize outcome reporting and prioritize individual participant data meta-analyses to address these gaps.

In conclusion, this meta-analysis demonstrates that MI supplementation improves the MII oocyte rate and the fertilization rate for women undergoing IVF. The uncertainty of the results should be considered with individual preferences when making clinical decisions. Further, clinical research and extensive multicenter randomized controlled trials needed to be conducted to assess its supplementation mode and better understand the working mechanism, establishing a more robust evidence base for clinical practice.

## Data Availability

The original contributions presented in the study are included in the article/**Supplementary Material**. Further inquiries can be directed to the corresponding author.
