# Supplementary figures and images for: Effect of myo-inositol supplementation in mixed ovarian response IVF cohort: a systematic review and meta-analysis
Source: Front Endocrinol (Lausanne). 2025 Mar 21;16:1520362. doi: 10.3389/fendo.2025.1520362 (PMC11968372; doi:10.3389/fendo.2025.1520362)

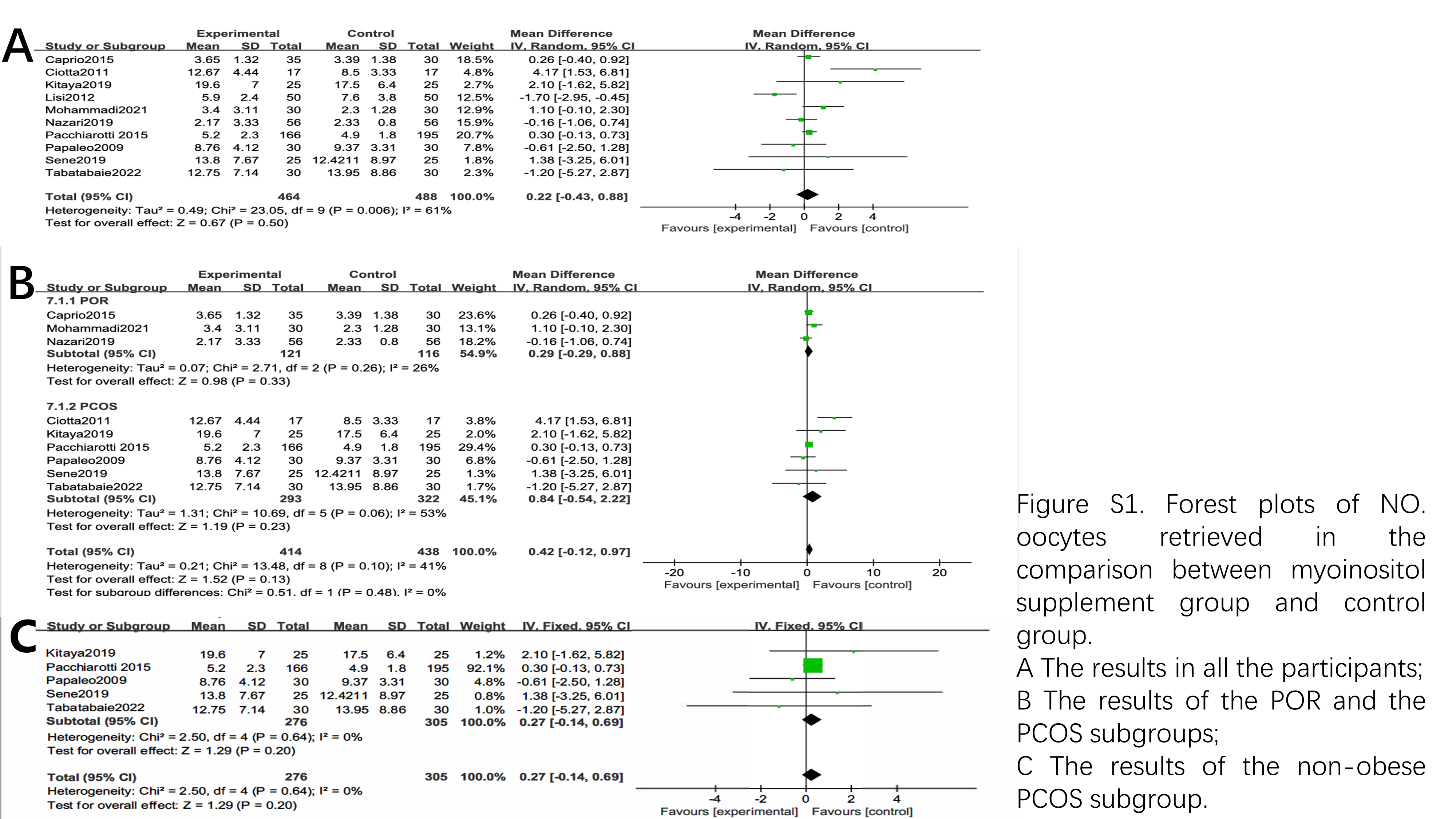

Supplement: Supplementary file 1 [file Image1.tif]

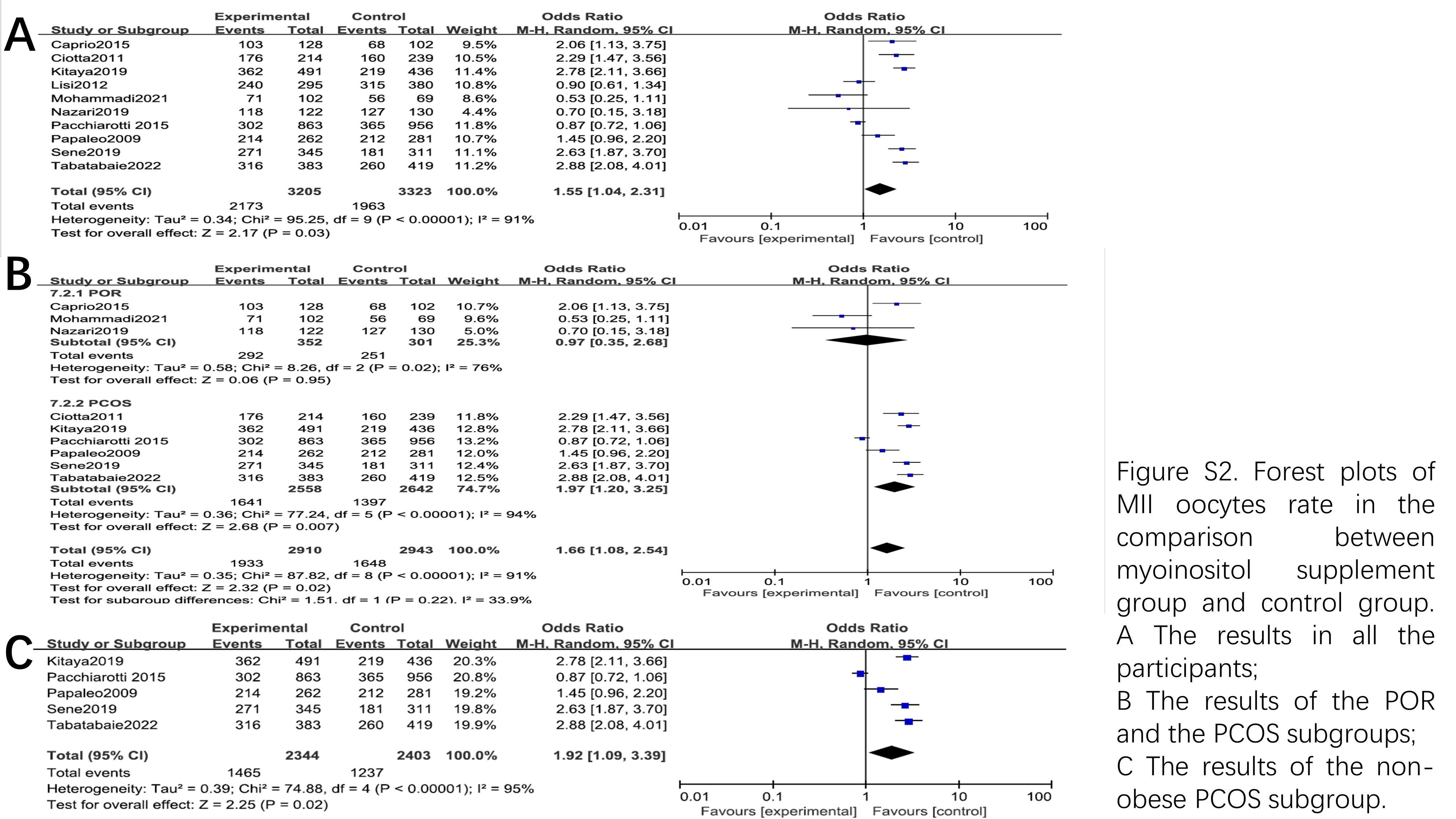

Supplement: Supplementary file 2 [file Image2.tif]

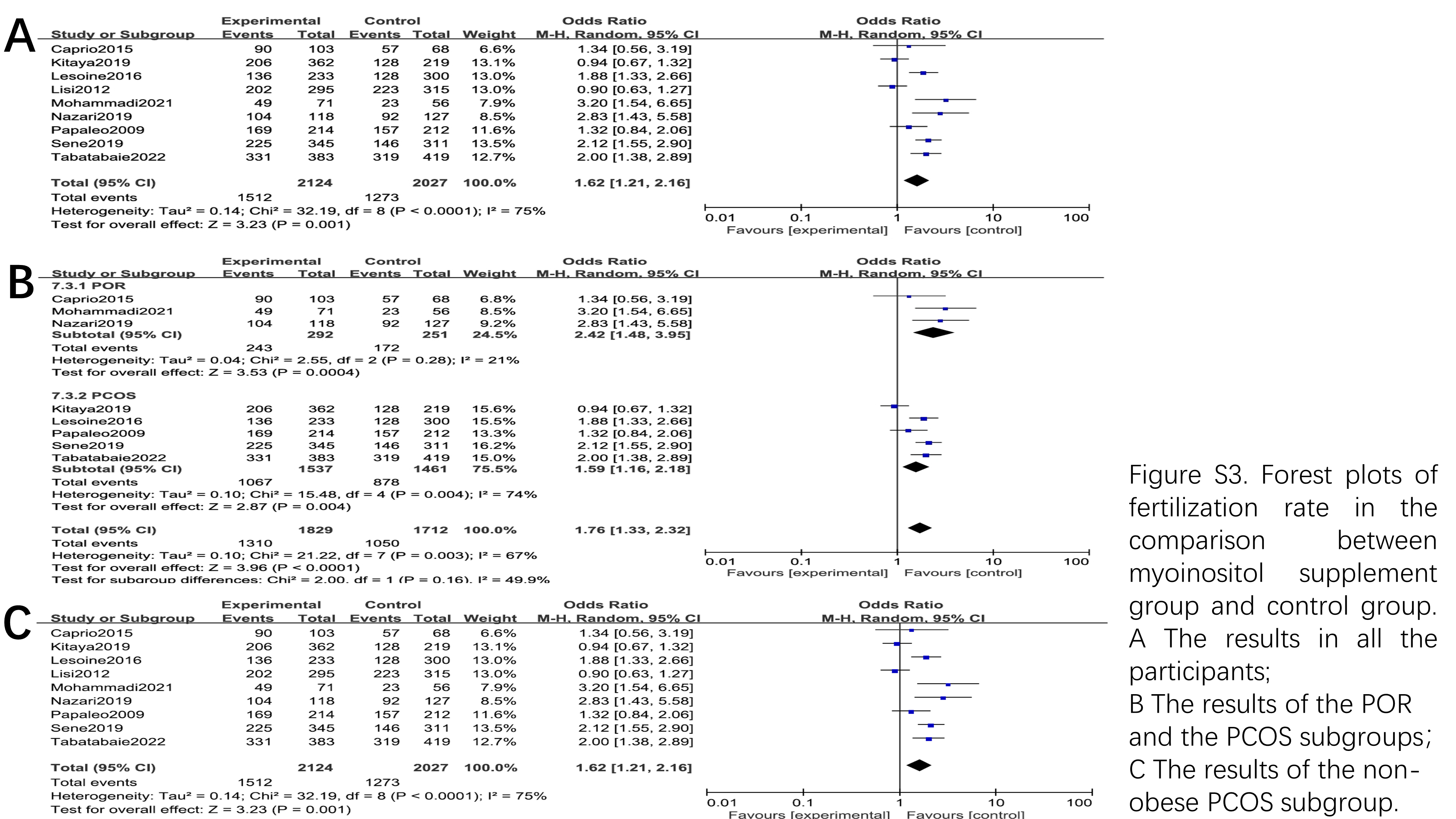

Supplement: Supplementary file 3 [file Image3.tif]

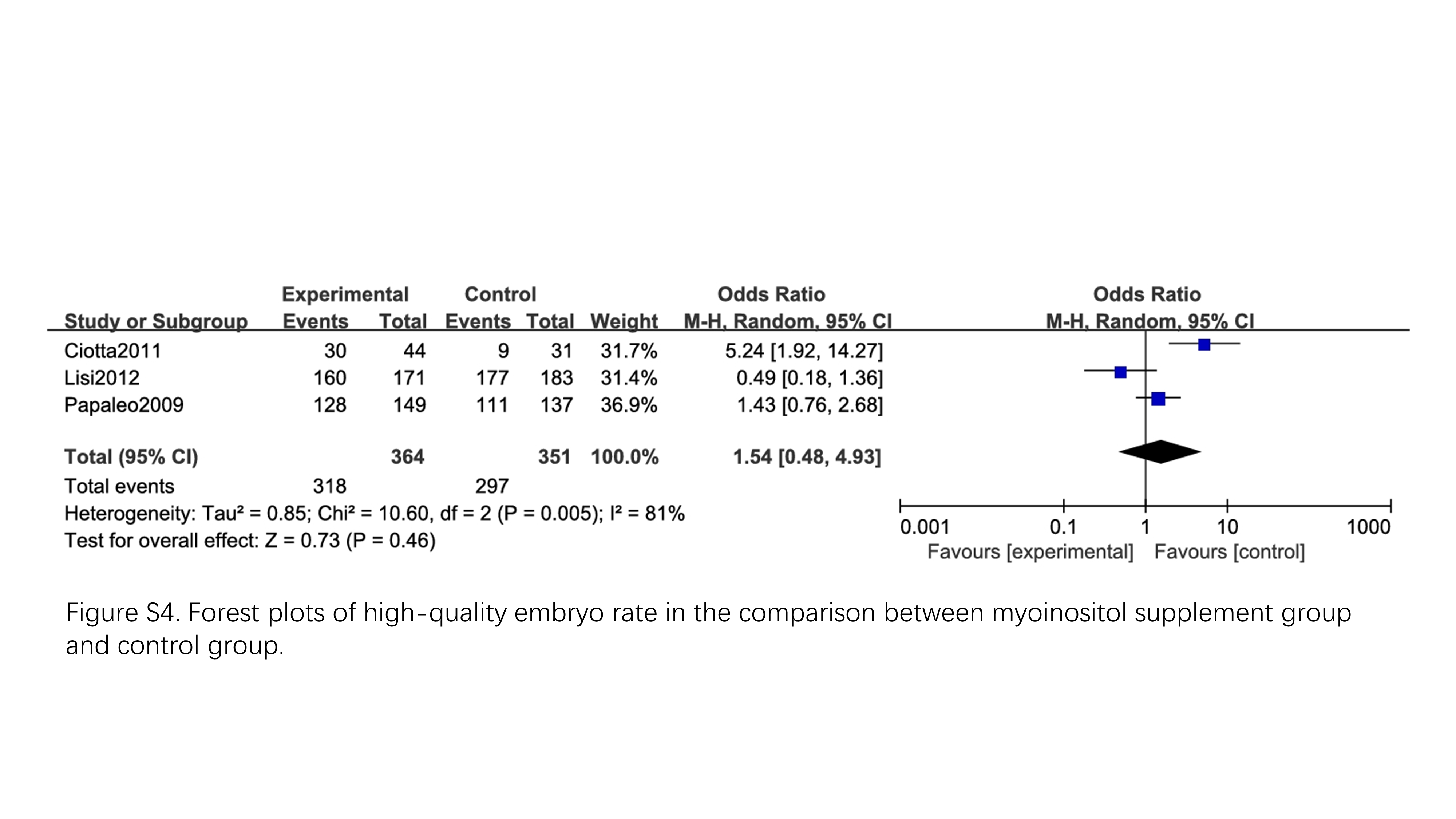

Supplement: Supplementary file 4 [file Image4.tif]

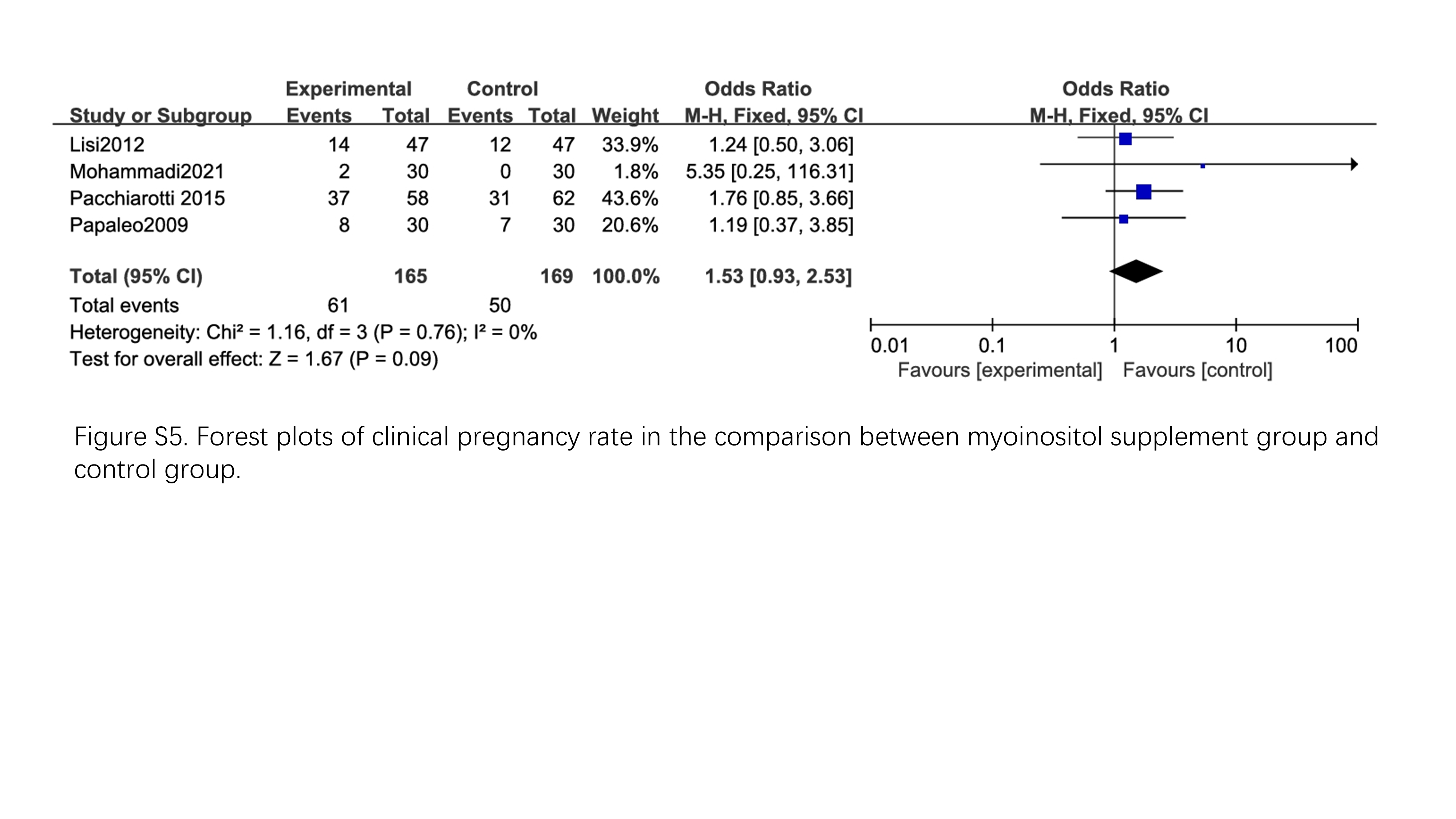

Supplement: Supplementary file 5 [file Image5.tif]
