# Supplementary material for: Effect of myo-inositol supplementation in mixed ovarian response IVF cohort: a systematic review and meta-analysis
Source: Front Endocrinol (Lausanne). 2025 Mar 21;16:1520362. doi: 10.3389/fendo.2025.1520362 (PMC11968372; doi:10.3389/fendo.2025.1520362)
Supplement: Supplementary file 6 [file Table1.docx]

| Included study | Randomization method | Allocation concealment | Blinding | Intention to treat and follow up |
| --- | --- | --- | --- | --- |
| Ciotta 2011 | 2 | 1 | 1 | 1 |
| Sene 2019 | 2 | 2 | 1 | 1 |
| Lesoine 2016 | 2 | 1 | 1 | 1 |
| Nazari 2019 | 2 | 1 | 1 | 1 |
| Mohammadi 2021 | 2 | 1 | 2 | 1 |
| Papaleo 2009 | 2 | 1 | 1 | 1 |
| Tabatabaie 2022 | 1 | 2 | 2 | 1 |
| Pacchiarotti 2015 | 2 | 1 | 2 | 2 |

Table S1. Quality assessment by Cochrane Handbook for Systematic Reviews of Interventions.
