# Supplementary material for: Effect of myo-inositol supplementation in mixed ovarian response IVF cohort: a systematic review and meta-analysis
Source: Front Endocrinol (Lausanne). 2025 Mar 21;16:1520362. doi: 10.3389/fendo.2025.1520362 (PMC11968372; doi:10.3389/fendo.2025.1520362)
Supplement: Supplementary file 7 [file Table2.docx]

| Included study | Selection | | | | Comparability | | Outcome | | | Total Stars |
| --- | --- | --- | --- | --- | --- | --- | --- | --- | --- | --- |
|  | Representativeness of the exposed cohort | Selection of the non exposed cohort | Ascertainment of exposure | Demonstration that outcome of interest was not present at start of study | Study controls for the most important factor | Study controls for second important factor | Assessment of outcome | Was follow-up long enough for outcomes to occur | Adequacy of follow up of cohorts |  |
| Caprio  2015 | 1 | 1 | 1 | 1 | 1 | 1 | 1 | 1 | 1 | 9 |
| Kitaya  2019 | 1 | 1 | 1 | 1 | 1 | 0 | 1 | 0 | 0 | 7 |
| Lisi  2012 | 1 | 1 | 1 | 1 | 1 | 1 | 1 | 0 | 1 | 8 |

Table S2. Quality assessment by Newcastle–Ottawa Scale.
